# Hemodynamic Evaluation of the Right Heart-Pulmonary Circulation Unit in Patients Candidate to Transjugular Intrahepatic Portosystemic Shunt

**DOI:** 10.3390/jcm11020461

**Published:** 2022-01-17

**Authors:** Giulia Manguso, Anthony Vignone, Manuela Merli, Cristiano Miotti, Annalisa Caputo, Carmine Dario Vizza, Roberto Badagliacca

**Affiliations:** 1 Department of Cardiovascular and Respiratory Science, Sapienza University of Rome, 00161 Rome, Italy; cristiano.miotti@uniroma1.it (C.M.); annalisa.caputo@uniroma1.it (A.C.); dario.vizza@uniroma1.it (C.D.V.); roberto.badagliacca@uniroma1.it (R.B.); 2 Department of Translational and Precision Medicine, Sapienza University of Rome, 00161 Rome, Italy; anthony.vignone@uniroma1.it (A.V.); manuela.merli@uniroma1.it (M.M.)

**Keywords:** portal hypertension, transjugular intrahepatic portosystemic shunt, heart failure, pulmonary hypertension, right heart catheterization, fluid challenge

## Abstract

In Europe, liver cirrhosis represents the fourth-most common cause of death, being responsible for 170,000 deaths and 5500 liver transplantations per year. The main driver of its decompensation is portal hypertension, whose progression radically changes the prognosis of affected patients. Transjugular intrahepatic portosystemic shunt (TIPS) is one of the main therapeutic strategies for these patients as it reverts portal hypertension, thus improving survival. However, the coexistence of portal hypertension and pulmonary hypertension or heart failure is considered a contraindication to TIPS. Nevertheless, in the latest guidelines, the definition of heart failure has not been specified. It is unclear whether the contraindication concerns the presence of clinical signs and symptoms of heart failure or hemodynamic changes in the right heart-pulmonary circulation. Moreover, data about induced right heart volume overload after TIPS and the potential development of heart failure and pulmonary hypertension is currently scanty and controversial. In this article we revise this issue in finding predictors of cardiac performance after TIPS procedure. Performing a fluid challenge during right heart catheterization might be a promising expedient to test the adaptation of the right ventricle to a sudden increase in preload in the first few months after TIPS. This test may unmask a potential cardiac inability to sustain the hemodynamic load after TIPS, allowing for a clearer definition of heart failure and, consequently, a more robust indication to TIPS.

## 1. Portal Hypertension: Epidemiology and Clinic Impact

In Europe, liver cirrhosis is the cause of 5500 liver transplantations per year and it constitutes the fourth most-common cause of death, resulting in 170,000 deaths per year [1]. The leading cause of morbidity and mortality in patients affected by liver cirrhosis is portal hypertension [2], which ensues/occurs progressively during the course of the disease [3].

Portal hypertension is a syndrome caused both by an increased resistance in the portal intrahepatic circulation and by increased splanchnic blood flow. In healthy liver, the pressure difference between the portal and the hepatic veins does not usually exceed 5 mmHg. Portal hypertension is defined by a gradient greater than 6 mm Hg, even if clinical complications do not seem to occur until the gradient exceeds 10–12 mmHg [4]. The pressure increase beyond this threshold is defined as clinically significant portal hypertension (CSPH). At this stage, patients usually have gastroesophageal varices and/or ascites. Later on, decompensated cirrhosis is characterized by episodes of variceal hemorrhage, intractable ascites, hepatorenal syndrome, or hepatic encephalopathy that may occur separately or in association [5,6,7]. Patients with CSPH reach the decompensated stage at a rate of 5–7% per year [8]. This is a fundamental transition in the natural history of cirrhosis because of its predictive role in survival. Hepatic decompensation reduces median survival from more than 12 years to about 2 years [8]. Patients with hepatic decompensation commonly have a longer hospital stay and a 10–20% risk of in-hospital death [7]; the portohepatic gradient is an independent prognostic factor, with a 3% increase in mortality for each 1 mmHg of gradient increase [9]. In decompensated stages, the main therapeutic goals are the reduction of mortality by preventing further decompensations/complications and, simultaneously, the prevention of acute-on-chronic liver failure [5]. In advanced liver disease the only definitive therapy is liver transplantation (LT) [10]. It is recommended to list patients for LT for a Model for End-Stage Liver Disease (MELD) score ≥ 15, based on creatinine, bilirubin and international normalized ratio [11]. While there is a general consensus on the criteria for LT, organ shortage is frequently a limiting factor, leading to increased rates of patient morbidity and mortality while on the waiting list [12].

## 2. Transjugular Intrahepatic Portosystemic Shunt (TIPS): Patient Selection and Impact on Hemodynamics

The introduction of transjugular intrahepatic portosystemic shunt (TIPS) in clinical practice represented, in the last 20 years, a step forward for treatment improvement for patients with untreatable complications of portal hypertension.

TIPS works as a shunt between the systemic and the portal circulation. It is obtained by percutaneously connecting an intra-parenchymal branch of the portal vein with a hepatic vein through a low-resistance conduit, embraced by a self-expandable metal stent. Thus, TIPS reduces the portal–caval pressure gradient. The current criteria for TIPS placement have been enlarged [13]. Clinical indications for TIPS include acute variceal bleeding refractory to treatment, recurrent or refractory ascites, refractory hepatic hydrothorax, hepatorenal syndrome, non-cirrhotic portal hypertension (Budd-Chiari syndrome, porto-sinusoidal vascular disease). In recent years, the role of pre-emptive TIPS has been proposed in selected patients with varices at high risk of bleeding and in patients with recurrent but not refractory ascites [14]. Contraindications to TIPS placement include significant pulmonary hypertension, heart failure or severe cardiac valvular insufficiency, rapidly progressive liver failure, severe or uncontrolled hepatic encephalopathy, uncontrolled systemic infection or sepsis, unrelieved biliary obstruction, polycystic liver disease and extensive primary or metastatic hepatic malignancy [15].

The impact of TIPS on pulmonary hemodynamics is still poorly discussed in the scientific literature. Creation of a TIPS may critically influence the pulmonary circulation. Blood shifts from the splanchnic circulation to the systemic circulation, thus increasing cardiac preload. Such changes can be clearly evidenced utilizing invasive hemodynamic techniques through right heart catheterization. Indeed, blood shunting results in an increase in cardiac output (CO) and pulmonary artery pressure and a decrease in systemic vascular resistances [16]. A recent study has shown that CO increases by 22% and systemic vascular resistance decreases by 26% after TIPS placement. Similarly, right atrial pressure (RAP) and mean pulmonary artery pressure (mPAP) may increase by 50 and 40%, respectively [17]. The study by Modha et al. has shown a tight correlation between the pre-TIPS right atrial pressure and the onset of symptomatic heart failure after TIPS placement [18].

Finally, it was shown that volume overload induced by TIPS placement may be associated with increased PAWP and, consequently, postcapillary pulmonary hypertension (group II WHO classification) [17,19,20,21,22,23,24] (Table 1).

## 3. Current Guidelines for TIPS Implantation

Cardiac assessment for elective TIPS insertion, according to guidelines [15], includes the evaluation of cardiac history, clinical examination, 12-lead ECG and N-Terminal pro-B-type natriuretic peptide (NT-proBNP) (strong recommendation, moderate-quality evidence). Further cardiac evaluation (echocardiogram +/− cardiology consultation) should be undertaken before elective TIPS if any of these are abnormal (strong recommendation, moderate-quality evidence).

TIPS is contraindicated in patients with severe left ventricular dysfunction or severe pulmonary hypertension (strong recommendation, moderate-quality evidence) [15]. However, guidelines on TIPS by the Clinical Services and Standards Committee (CSSC) of the British Society of Gastroenterology (BSG) limit the definition of severe pulmonary hypertension to qualitative assessment, not considering a more specific invasive hemodynamic evaluation. Furthermore, severe left ventricular dysfunction has not been defined by clinical or hemodynamic parameters. Therefore, it remains unclear whether the heart failure contraindicates TIPS in the presence of clinical sign and symptoms or including hemodynamic impairment.

On the other hand, a Consensus Conference on TIPS management published in 2017 proposed hemodynamic and echocardiographic thresholds contraindicating TIPS positioning in case of pulmonary hypertension (mPAP > 45 mmHg at RHC, sPAP > 50 mmHg at echocardiography). These thresholds, indicative of severe pulmonary hypertension (PH), would be predictive of cardiac dysfunction after TIPS. However, TIPS can be performed in mild PH (mPAP at RHC between 25 and 35 mmHg) [32]. These data have apparently not been considered in the latest guidelines. Surprisingly, the former Consensus Conference did not allow further identification of thresholds for left heart failure [32].

Even if there is a growing awareness of the importance of a correct cardiac evaluation, since blood shunting after TIPS is responsible for a consistent increase in right atrial pressure (RAP) and cardiac output (CO), predictive studies of adverse cardiac outcomes after TIPS are currently scarce and controversial [15]. A study worth mentioning by Billey et al. identified the presence of one of the following features as predictive factors for cardiac decompensation after TIPS implantation: prolonged QT interval, elevated BNP or NT-proBNP, left atrial dilatation, elevated E/A and E/e’ (early maximum ventricular filling velocity/atrial maximal ventricular filling velocity, early diastolic transmitral flow velocity/early diastolic mitral annulus velocity), and aortic stenosis. The proposed Toulouse algorithm, aimed at reducing the occurrence of heart failure after TIPS, with a two-step approach moving from BNP or NT-proBNP to echocardiographic assessment of left heart chambers. The study showed a high proportion of patients with heart failure after TIPS implantation (20% of patients). However, the study was not statistically sound and the proposed algorithm needs further validation [25]. A high proportion of patients with left ventricular diastolic dysfunction has also been reported after TIPS placement [33].

The proportion of patients with possible cardiac complications may further increase in the near future as patients with cirrhosis of metabolic origin are increasing in prevalence. These patients show concomitant diabetes, arterial hypertension, and/or high HDL cholesterol or triglycerides concentration in blood, and increased cardiovascular risk. For this reason, an accurate cardiac assessment becomes crucial in their evaluation before TIPS placement [34]. The correct evaluation of the right heart-pulmonary circulation unit could also be useful for the assessment of patient candidates for pre-emptive TIPS, where risk stratification appears to have a major impact on outcome [35].

Unfortunately, long-term changes in the pulmonary circulation-heart unit induced by volume overload after TIPS placement are currently overlooked in clinical practice [26,27,28,29,30,31,36].

## 4. Future Perspective: Should We Stress the Pulmonary Circulation?

The potential impact of TIPS placement on the pulmonary circulation offers the opportunity to better clarify the correct balance of its clinical indication.

According to current guidelines/consensus, increased right and/or left ventricular diastolic pressure would allow for TIPS contraindication even in the absence of structural heart diseases [13,14]. Indeed, as previously mentioned, portal hypertension is usually associated with volume overload of the pulmonary circulation-heart unit [17,19,20,21,22,23,24]. For this reason, right heart catheterization should be mandatory for the initial assessment of patient candidates for TIPS. Limiting the evaluation of ventricular filling pressure to echocardiography-derived diastolic function parameters, as suggested by some authors [25], is known to be associated with low accuracy, leading to unacceptable false-positive and false-negative rates [37].

Invasive hemodynamic assessment would also allow us to clearly identify increased pulmonary pressure, which represents a second important contraindication for TIPS placement. The correct measurement of PAWP allows definitively to identify precapillary pulmonary hypertension (mPAP > 20 mmHg, PAWP ≤ 15 mmHg, PVR ≥ 3 WU) from postcapillary pulmonary pressure (mPAP > 20 mmHg, PAWP > 15 mmHg, PVR < 3 WU) [38,39].

Additionally, RHC assessment gives us the unique opportunity to test the potential consequences of TIPS-derived volume overload on the pulmonary circulation-heart unit.

Considering such important clinical consequences of TIPS placement, a fluid challenge during initial RHC could be a promising tool to correctly evaluate the potential hemodynamic changes induced in the pulmonary circulation-heart unit in patient candidates for TIPS.

In recent years, indeed, many studies have shown that a fluid challenge with rapid saline infusion during RHC was useful to correctly identify hidden left heart failure related to left ventricular diastolic dysfunction in the absence of structural heart diseases [40]. Fluid challenging the pulmonary circulation-heart unit would allow us to correctly identify those patients at high risk of increased LV filling pressure (i.e., PAWP) after TIPS implantation leading to heart-failure-symptoms development. Similarly, fluid challenging a failing right ventricle would be associated with increased right atrial pressure (RAP) with little increase in CO. A transient increase in systemic venous return would definitively allow us to test right ventricle adaptation to volume overload, identifying those patients at risk of right heart failure development after TIPS placement. In contrast, a normal right ventricle would respond with an increase in CO and unchanged RAP [41].

The fluid challenge test during initial RHC could help clinicians in the difficult task of deciding on TIPS indication in a different clinical setting. A relevant cut-off value of RAP increase during rapid saline infusion would probably improve our understanding for the correct balance between indication and contraindications to TIPS placement and provide essential information on outcomes for future decision-making processes.

Further investigations on pulmonary circulation-heart unit behaviors and their influence on the outcome of TIPS are still required.

## Figures and Tables

**Table 1:** Cardiac complications related to transjugular intrahepatic portosystemic shunt.

Study Design	Author	Patients N	Event	Methods
Prospective	Billey et al. [25]	111	Hospitalization for heart failure, 20%	Clinical
Retrospective	Busk et al. [17]	13	RAP increase, % not reported mPAP increase, % not reported Heart rate increase, % not reported Cardiac output increase, % not reported	Right heart catheterization
Prospective	Merli et al. [26]	11	Increase in LVDD at 12 months, % not reported	Echocardiography
Retrospective	Modha K et al. [18]	882	Symptomatic heart failure, 0.8%	Clinical
Prospective	Radunski UK et al. [27]	16	Increase in LVDD at 6 months, % not reported	Magnetic resonance
Prospective	Pudil et al. [28]	49	LVDD increase at 6 months, % not reported	Echocardiography
Retrospective	Kovács A. et al. [29]	11	Increase in LVEDV at 24 h, % not reported	Magnetic resonance
Retrospective	Parvinian A. et al. [30]	125	Increase in RA at 90 days, % not reported	Echocardiography
Prospective	Trevisani F. et al. [31]	29	Prolonged QT interval, 80%	Electrocardiogram

RAP: right atrial pressure; mPAP: mean pulmonary arterial pressure; LVDD: left ventricular diastolic diameter; LVEDV: left ventricular end-diastolic volume.
